# Treatment of intestinal tuberculosis with small bowel perforation: a case report

**DOI:** 10.1186/s13256-021-02752-2

**Published:** 2021-03-31

**Authors:** Daniel Sasse, Christoph D. Spinner, Kathrin Rothe, Jochen Schneider, Jochen Gaa, Silvia Würstle

**Affiliations:** 1 Department of Radiology, School of Medicine, University Hospital rechts der Isar, Technical University of Munich, Munich, Germany; 2 Department of Internal Medicine II, School of Medicine, University Hospital rechts der Isar, Technical University of Munich, Munich, Germany; 3 School of Medicine, Institute for Medical Microbiology, Immunology and Hygiene, University Hospital rechts der Isar, Technical University of Munich, Munich, Germany

**Keywords:** Intestinal tuberculosis, *Mycobacterium tuberculosis*, Small bowel perforation

## Abstract

**Background:**

Diagnosis of intestinal tuberculosis poses a dilemma to physicians due to nonspecific symptoms like abdominal pain, fever, nausea, and a change in bowel habit. In particular, the distinction between inflammatory bowel disease and intestinal tuberculosis remains challenging.

**Case presentation:**

A 27-year-old man from Colombia presented with fever, night sweats, and progressive lower abdominal pain. Computed tomography revealed a thickening of the bowel wall with a mesenterial lymphadenopathy, ascites ,and a pleural tumor mass. Histology of intestinal and pleural biopsy specimens showed a granulomatous inflammation. Although microscopy and polymerase chain reaction (PCR) for *Mycobacterium tuberculosis* (MTB) were negative, empirical MTB treatment was initiated on suspicion. Due to a massive post-stenotic atrophied intestinal bowel, MTB medications were administered parenterally in the initial phase of treatment to guarantee adequate systemic resorption. The complicated and critical further course included an intra-abdominal abscess and bowel perforation requiring a split stoma, before the patient could be discharged in good condition after 3 months of in-hospital care.

**Conclusions:**

This case highlights the clinical complexity and diagnostic challenges of intestinal MTB infection. A multidisciplinary team of physicians should be sensitized to a timely diagnosis of this disease, which often mimics inflammation similar to inflammatory bowel disease, other infections, or malignancies. In our case, radiological findings, histological results, and migratory background underpinned the suspected diagnosis and allowed early initiation of tuberculostatic treatment.

## Background

It is hypothesized that almost one-quarter of the global population is latently infected with *Mycobacterium tuberculosis* (MTB) [1]. In 5–10% of cases, latent tuberculosis proceeds to active tuberculosis disease. Due to the specific characteristics of mycobacteria, which are acid-fast, very slow growing, and able to persist within macrophages, the clinical appearance differs markedly from other bacterial infectious diseases. Abdominal MTB ranks only sixth in extrapulmonary MTB manifestations and presents mostly as peritoneal MTB [2, 3]. In 2018, 98 cases of intestinal MTB were reported in Germany, corresponding to a local annual incidence of approximately 1 in 1,000,000 [4].

## Case presentation

**Fig. 1:**
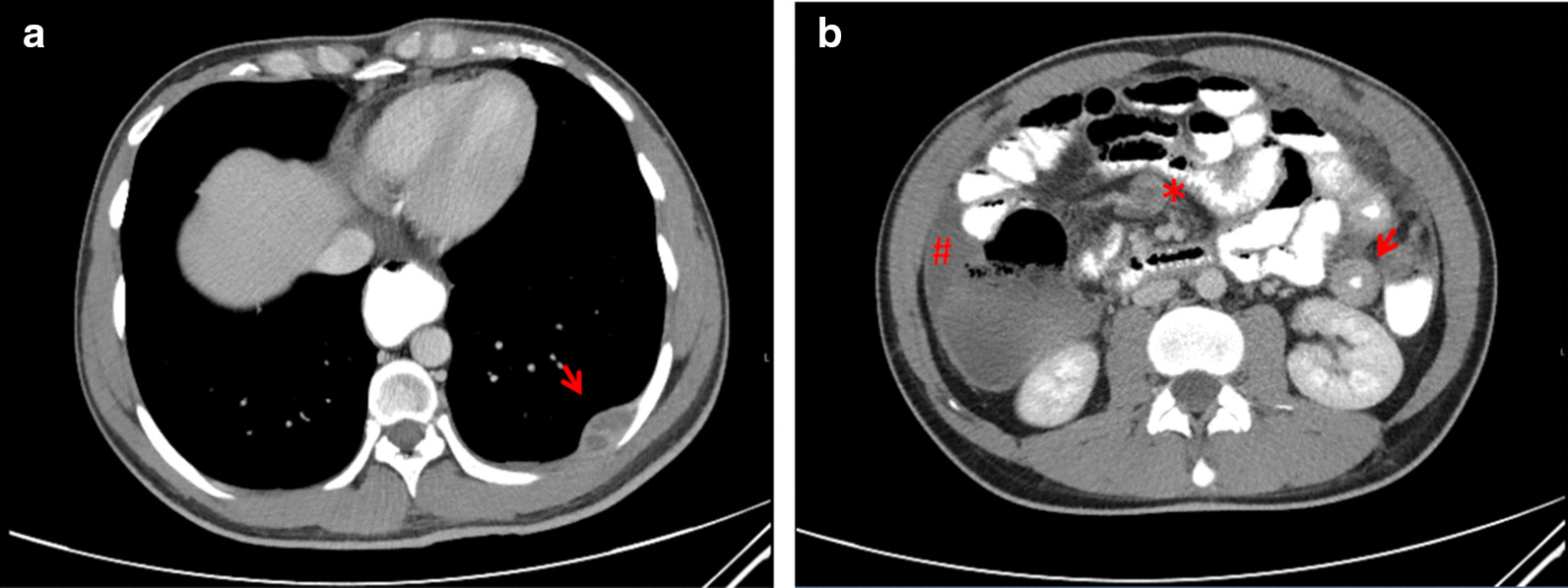
Initial computed tomography scan demonstrating (**a**) a pleural mass (the distal esophagus is distended as a secondary finding) and (**b**) necrotic abdominal lymph nodes (*), bowel wall thickening (→), and ascites (#)

A 27-year-old man from Colombia presented to our emergency department with fever (38.9 °C) and progressively worsening lower abdominal pain for the last 6 months, peaking at the day of admission. The patient also reported mild nausea, intermittent night sweats, weight loss, and fever during the last few months prior to admission. Physical examination revealed no noticeable findings aside from pressure pain in the lower right abdominal region. Laboratory inflammatory markers were elevated (leukocyte count 13 g/L, C-reactive protein [CRP] 17 mg/dL). The patient was admitted to our hospital. Due to findings of ascites in an ultrasound examination, a computed tomography (CT) scan was performed and revealed a pleural mass, necrotic abdominal lymph nodes, and bowel wall thickening (Fig. 1).

A computed tomography (CT)-guided biopsy of the pleural tumor was performed. While histopathological results were still pending, the patient developed increasing abdominal pain and vomiting, so another CT scan was conducted, which showed a small bowel ileus. Subsequently, a laparoscopy with peritoneal biopsy and creation of a protective ileostomy was performed. The histological examination of the pleural and abdominal specimens showed granulomatous inflammation without direct microscopic detection of acid-fast bacilli. Polymerase chain reaction (PCR) for *M. tuberculosis* complex from pleural and intra-abdominal specimens revealed a negative result.

As intestinal malabsorption was assumed due to a massive post-stenotic atrophied intestinal bowel, parenteral nutrition and empirical intravenous MTB treatment with ethambutol, levofloxacin, rifampicin, and isoniazid was initiated using a totally implantable venous access device (port). After 5 weeks, MTB cultures of pleural biopsy and intra-abdominal lymph nodes showed growth of a pan-sensitive *M. tuberculosis* complex strain.

In the further in-hospital course, the patient developed severe abdominal pain in the region of the ileostomy as well as high inflammatory markers. A CT scan displayed free air within the peritoneal cavity indicating a bowel perforation and an intra-abdominal abscess, leading to the performance of a laparotomy with small bowel segment resection and preparation of a split stoma. Two months later, the split stoma was reversed. After 3 months of in-patient care, the patient was discharged in good condition, and MTB treatment was switched to oral therapy with isoniazid and rifampicin. After a total duration of 10 months, a CT scan showed no evidence of persistent MTB disease, so the MTB treatment was terminated.

## Discussion

We demonstrate a case of abdominal MTB with small bowel affection resulting in a complicated course with a small bowel ileus, massive post-stenotic atrophied intestine, and consecutive small bowel perforation with intra-abdominal abscess requiring segmental bowel resection. The patient was discharged after 3 months of in-patient care and finished tuberculostatic therapy after 10 months.

Due to the limited function of the post-stenotic intestinal bowel tract, oral nutrition had to be replaced by parenteral nutrition. Simultaneously, anti-tuberculosis treatment was administered intravenously via a port. In the present case, parenteral administration was switched to oral therapy after 12 weeks.

The complications of this case—an ileus and ileum perforation with intra-abdominal abscess—both required surgery. In 2017, a study on intestinal tuberculosis showed a complication rate of 44 % (27/61), including formation of abscesses, fistulas, strictures, perforation, and obstruction [5]. Lee *et al*. reported a perforation rate of 6.6 % (6/91) in abdominal MTB [6]. In that study, one of six patients with perforation died, whereas other authors indicate a higher mortality rate of 25 % following tubercular bowel perforations [6, 7]. Therefore, to guarantee rapid diagnosis and treatment of these life-threatening complications, close cooperation between infectious disease specialties, radiology, and surgery is essential.

Severe complications of intra-abdominal tuberculosis can be avoided if adequate treatment is provided in a timely manner. However, early diagnosis of intra-abdominal tuberculosis is challenging. Diagnosis of abdominal MTB is often hampered by nonspecific symptoms like abdominal pain accompanied by nausea and a change in bowel habits [5]. Less frequent symptoms are dyspepsia, anal strictures, or fistulas [8]. Radiological findings are mostly nonspecific, as intestinal tuberculosis can mimic inflammation, infection, or malignancies [9]. In particular, the distinction between inflammatory bowel disease and abdominal MTB infection remains a diagnostic challenge [10].

Contrast-enhanced CT is the recommended imaging tool in abdominal MTB. Abdominal lymphadenopathy and short-segment strictures with symmetrical concentric wall thickening with homogeneous contrast enhancement are the most common findings [11], while heterogeneous asymmetrical and focal thickening is usually associated with malignant neoplasia [12]. Infiltration of the mesenteric fat is associated more with Crohn’s disease than with abdominal MTB infection [13].

In patients with acute complications of abdominal MTB, evidence of pulmonary MTB infection on chest X-rays may be absent. Varying rates have been reported, with pulmonary findings in 39–80% [13, 14].

Aside from clinical and radiological difficulties, microbiological confirmation of diagnosis poses an additional challenge. MTB culture is considered the gold standard due to possible false-negative results of microscopy and PCR. However, abdominal MTB is usually a paucibacillary infection, so it can take several weeks to get a positive culture result. In the current case, the first intra-abdominal MTB culture was positive after 5 weeks of incubation, but MTB treatment had been commenced on suspicion.

Hence, a histological result of granulomatous inflammation, radiology, and the migratory background are decisive in establishing the correct diagnosis in a timely manner when microbiology is still pending.

## Conclusion

Bowel wall perforations due to intestinal MTB are associated with high mortality, thus requiring rapid diagnosis and therapy. However, diagnosis of abdominal MTB is challenging due to nonspecific clinical and radiological signs and its paucibacillarity, which reduces the sensitivity of microbiological methods. Impaired bowel function by *M. tuberculosis* might lead to complications as presented in this case such as ileus or perforation, as well as malabsorption requiring parenteral administration of anti-tuberculosis therapy. A combined approach of surgery and tuberculostatic therapy led to complete cure of this highly unique complicated case.

## Data Availability

Not applicable.

## Abbreviations

CRP: C-reactive protein
CT: Computed tomography
MTB: *Mycobacterium tuberculosis*
PCR: Polymerase chain reaction
TB: Tuberculosis
